# Comparing the Suitability of Autodock, Gold and Glide for the Docking and Predicting the Possible Targets of Ru(II)-Based Complexes as Anticancer Agents

**DOI:** 10.3390/molecules18043760

**Published:** 2013-03-25

**Authors:** Adebayo A. Adeniyi, Peter A. Ajibade

**Affiliations:** Department of Chemistry, University of Fort Hare, Private Bag X1314, Alice 5700, South Africa

**Keywords:** docking, meta-based complexes, binding site interaction, unusual ligands, cancer receptors

## Abstract

In cancer chemotherapy, metal-based complexes have been recognized as the most promising means of inhibiting cancer growth due to the successful application of *cis*-platin and its derivatives above many of the existing organic anticancer agents. The limitations in their rational design can be traced to the complexity of the mechanism of their operations, lack of proper knowledge of their targets and lack of force fields in docking packages to appropriately define the metal centre of the organometallic complexes. In this paper, some of the promising anticancer complexes of Ru(II) such as the rapta-based complexes formulated as [Ru(η^6^-*p*-cymene)L_2_(pta)] and those with unusual ligands are considered. CatB and kinases which have been experimentally confirmed as possible targets of the complexes are also predicted by the three methods as one of the most targeted receptors while TopII and HDAC7 are predicted by two and one of the methods as best targets. The interesting features of the binding of the complexes show that some of the complexes preferentially target specific macromolecules than the others, which is an indication of their specificity and possibility of their therapeutic combination without severe side effects that may come from competition for the same target. Also, introduction of unusual ligands is found to significantly improve the activities of most of the complexes studied. Strong correlations are observed for the predicted binding sites and the orientation of the complexes within the binding site by the three methods of docking. However there are disparities in the ranking of the complexes by the three method of docking, especially that of Glide.

## 1. Introduction

There are many promising potential anticancer drugs, especially ruthenium complexes with better activities against cancer than the most commonly used platinum chemotherapies, but their targets remain unknown [1,2,3,4,5,6,7,8,9]. There are clear evidences that other target proteins are the most likely targets for organometallic anticancer complexes other than DNA, which is an established *cis*-platin target. Ruthenium antitumor agents generally display lower reactivity towards double-stranded DNA and their cellular mechanisms of action are not known [10]. The complexes of the type [Ru(η6-p-cymene)L2(pta)] (where pta is 1,3,5-triaza-7-phosphaadamantane), called rapta complexes, have been shown to have moderate anticancer activity in various cell lines and an excellent activity with regard to reducing the number and weight of solid metastases, but do not affect the primary tumour [8,11]. Some of the ruthenium-arene complexes have also been reported to have complicated ligand exchange chemistry and to be kinetically unstable [2]. Out of many derivatives of rapta that have been identified, synthesized and characterized, [Ru(η6-p-cymene)Cl_2_(pta)] called rapta-C (Table 1) has been pointed out to be the best anticancer complex [8]. A simple modification in the geometry of these molecules has been reported to lead to a significant change in their anticancer activities just as [Ru(η6-C_6_H_5_CF_3_)(pta)Cl_2_] with the electron-withdrawing α,α,α-trifluorotoluene ligand called rapta-T-CF_3_ is reported as another most cytotoxic complex of rapta, especially in A2780 human ovarian cancer cells and also significantly more cytotoxic than other simple rapta complexes [12]. 

Some of the different models of rapta complexes that have been reported in the literature [8,11,12] are used in this study with other proposed structures, especially the unusual metal-based complexes. Based on the available literature cited below and in an effort to enhance the activities of rapta complexes, we decided to consider some unusual ligand metal-based complexes in order to understand the possible changes in the activities of some known metal-based complexes of Ru(II). We suggested the hydroxide metal complexes of ruthenium (OH)n-Ru-Ln. Even though OH ion is mostly known to act as a bridge in metal-based complexes, there have also been reported cases in which the hydroxide ion is used as a nucleophilic ligand directly with a single metal [13,14]. Also, hydrogen as a hydride is generally known to act as a bridge but there have been reported cases of metal complexes where it is directly coordinated with single metals [15,16], and there have even been structures of ruthenium-based complexes with hydride [17]. It is also reported possible to have metal-carboxylate complexes as monodentate ligands [18] instead of the more commonly reported bidentate nature of the carboxylate ion [18,19]. Other models of unusual metal complexes considered are the [Ln-M-NH_2_] complexes. The common metal complexes of this type are ammonia-metal complexes [Ln-M-NH_3_], but the possibility of having amine-metal complexes [Ln-M-NH_2_] has also been reported [20]. Despite the high volume of the *in vitro*, *in vivo* and theoretical studies on the behaviour of the potential rapta anticancer drugs, the mechanism of action of these new complexes is not well known [21], consequently affecting the possibility of enhancing their antitumor effectiveness. Based on the contradiction between several strong *in vivo* activities and weak *in vitro* activities of novel anticancer derivatives of rapta, the poor correlation between the binding of rapta complexes to DNA and their cytotoxicity suggested that these potential anticancer drugs act through a mechanism different from the classical platinum anticancer drugs [21]. It has also been observed that the presence of the metal in complexes results in enhancement and/or introduction of some pharmacological properties into ligands like pta, ethylenediamine, and many others which on their own are noncytotoxic ligands leading to compounds with significant anticancer activity [22].

Therefore, in order to predict the possible targets and reasons for some of the traceable ineffectiveness of these metal-based complexes, ten protein targets which are recombinant human albumin (rHA), thymidylate synthase (TS), ribonucleotide reductases (RNR), histone deacetylase (HDAC7), cathepsin B (CatB), topoisomerase II (Top II), thioredoxin reductase (TrxR), BRAF kinase and histone protein in nucleosome core particle (NCP) are used in this project either due to their reported roles in cancer growth or as transport agents that affect drug phamarcokinetic properties (e.g., rHA). Also, DNA gyrase was included to study the possibility of anticancer complexes also acting as antimalarial agents. A ruthenium complex of the quinolone compound called ofloxacin, which is an inhibitor of DNA gyrase, has also been screened as an anticancer agent [23]. There are experimental reports that have suggested CatB, TrxR [4], HP-NCP [10] and kinase [24] as possible targets of some of the complexes considered in the work. rHA was selected due to its significant role in the pharmacokinetic availability of a wide range of drugs, including metallodrugs and consequentially in determining their bioavailability and toxicology [25]. It is also observed to accumulate in solid tumors and, consequently, has been exploited as a drug-delivery system [26]. In addition, it can also play a divergent role, either in delivery of metal-based anticancer drugs to their cellular targets or in deactivating them even before reaching the target(s) [22]. The TS gene is a critical enzyme in maintaining a balanced supply of deoxynucleotides required for DNA synthesis and repair [27]. Therefore, its inhibition is correlated with chromosome damage and fragile site induction [27]. RNR is responsible for the synthesis of DNA from the corresponding building blocks of RNA [28]. HDAC7 are proteins that assist in the packaging of DNA into chromosomes and help in gene regulation through acetylation and deacetylation. HDAC7 is part of the mechanism for DNA transcription and therefore, its inhibition make it a drug target because without its function of removing acetylated groups, the signalling switches will become stuck in one position and lose their effectiveness [28]. CatB is an enzyme that is involved in cellular metabolism and it is implicated to take part in the tumour progression and metastasis processes which makes it a suitable target for the design of anti-metastatic drugs [21]. It has also been reported to play significant roles in glioma invasion, which is a complex primary brain disease of tumor invasion [29]. 

There have been several successful applications of molecular docking studies in rational drug design, but they have limited application to study metal complexes [4], mostly due to the lack of appropriate force fields to take care of metal atoms [30] and their relativity properties. Docking suites like Gold, Glide and some others can possibly take care of metal atom if it is part of the receptor and remained unbounded. Therefore, there are limited number of docking studies done where metal is part of the ligands [4]. Also, Autodock can only be used for metal if the parameters for the metal of interest can be incorporated into the parameter file of the package. One of the areas of research interest to us is applying docking method for the rational design of Ru-based anticancer complexes as reported in our previous work where the receptor interactions of selected rapta complexes are compared to a set of proposed models of Ru(II) complexes [31], which is quite different from the set of models considered in this work. In this project, we try to understand what could be the possible targets of some anticancer metal-based complexes of interest using three packages: Autodock, Gold and Glide, and to the best of our knowledge the binding of many of these complexes and the selected targets have not been reported in the literature. However, one of the criteria for any possible application of docking for metal-based complexes is to make sure the geometries of the complexes are optimized. In this research work, the geometries of all the ruthenium-based complexes were first optimized using Firefly 7.1.G [32]. During the optimization, the hybrid dft functional PBE0 was applied and a combination of the external basis set SBKJC VDZ ECP was applied on Ru, P and Cl where applicable, while the remaining atoms were computed using the 6-31G basis set.

## 2. Results and Discussion

In this work, we have presented the binding modes, the best possible targets and binding site interactions of Ru-based complexes with ten receptors using Glide, Autodock and Gold docking. Even though a little comparison of the ranking obtained from the three methods are considered but the interest is to understand and to predict the optimal orientation and conformation of the complexes embedded in a protein which is the primary objective of all the research efforts in the area of protein-ligand interactions, development of many different techniques and the associated software tools [33]. Also, being careful of the inherent inaccuracies in the calculated estimates of the binding energy by each of the different docking method, the binding energy are ranked within each package and a cross ranking is avoided since the values are calculated using different docking programs with different force-fields [34]. Also, the order of ranked best complexes are carefully handled paying attention to the fact that within the same method of docking, there are ranges of error. For instance the reliability of the order for Autodock is ~2.177 kcal/mol standard error [33,35]. The results of the docking presented in this work is the best binding results out of the favourably 20 predicted by Autodock, 10 predicted by Gold and of 26 predicted by Glide. The structures of all the metal-based complexes that were used in the docking study are presented in Table 1.

**Table 1:** The structures of the metal-based complexes.

No	Structure/Name	No	Structure/Name	No	Structure/Name	No	Structure/Name
1	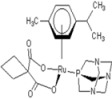 Carbo-rapta-C	7	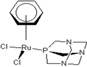 rapta-B	12	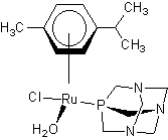 rapta-C-H_2_O	17	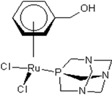 rapta-Ta-OH
2	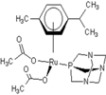 rapta-C-COOH	8	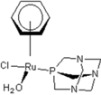 rapta-B-H_2_O	13	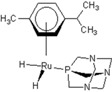 rapta-C-H	18	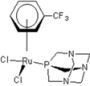 rapta-T-CF_3_
3	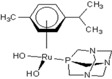 rapta-C-(OH)_2_	9	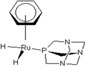 rapta-B-H	14	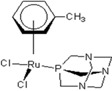 rapta-T	19	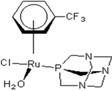 rapta-T-CF_3_(H_2_O)
4	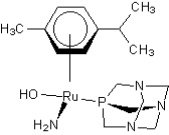 rapta-C-NH_2_(OH)	10	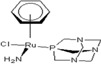 rapta-B-NH_2_	15	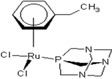 rapta-Ta-CH_3_	20	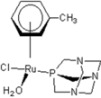 rapta-T-H_2_O
5	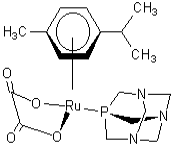 oxalo-rapta-C	11	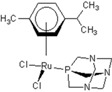 rapta-C	16	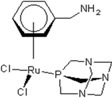 rapta-Ta-NH_2_	21	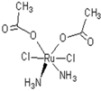 rClCOO-NH_3_
6	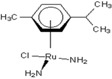 raC-NH_2_						

The general features from the Glide docking prediction (Table 2) show that the best predicted targets for most of the complexes are TopII, followed accordingly by kinase, RNR and CatB. Some of the complexes like **6**, **5**, **13**, **9**, **16** and **21**, respectively, bind to kinase preferentially compared to any other possible targets that are considered in this research. Also, **17**, **8** and **5** prefer to target TS than any other targets. Some of the least targeted receptors are rHA (with the exception of complex **21**), HDAC7, DNA gyrase, TrxR (except complex **21**). The preferred receptors that **1** is predicted to favourably target are HP-NCP, followed accordingly by CatB, TrxR and DNA gyrase. Those that are predicted to be rarely targeted by **1** are TopII, followed accordingly by rHA, TS, HDAC7 and RNR. However, based on Glide prediction, the complexes **8**, **13**, **17** and **20** bind most often favourably to most of the receptors than many of the other complexes considered. It became clear that hydrolysed complexes like **8**, **12**, **20** and **19** bind more tightly to the receptors than the parent complexes **7**, **11**, **14** and **18** due to activation by hydrolysis, which is in good agreement with the literature [3,12,22,36]. Also, modifications made to **14** such as in **16**, **17**, **18**, **19** significantly improved the binding of **14** on the studied receptors, except for **15**, which in many cases is poorer than the parent.

**Table 2:** The docking result of the metal-based complexes against ten receptors using the Glide package in Maestro.

		CatB	DNA gyrase	HDAC7	HP−NCP	KINASE	rHA	RNR	Top11	TrxR	TS
1	carbo-rapta-C	−2.14	−1.68	−1.41	−2.67	−3.45	−0.97	−1.51		−1.70	−1.00
2	rapta-C-COOH	−1.24	−1.67		−1.35			−0.88		−2.12	−1.22
3	rapta-C-(OH)_2_	−2.50	−2.14	−1.38	−3.02	−3.26	−1.49	−2.52	−3.12	−2.84	−1.91
4	rapta-C-NH_2_(OH)	−2.12	−1.88	−1.63	−1.99	−3.49		−1.16	−3.03	−2.44	−1.65
5	oxalo-rapta-C	−2.19	−2.19	−2.38	−2.15	−4.44	−2.12	−0.55		−2.69	−3.71
6	raC-NH_2_	−2.68	−2.51	−1.93	−3.56	−5.17	−2.91	−1.80	−3.44	−2.92	−2.93
7	rapta-B	−3.59	−2.53	−2.30	−2.84	−3.20	−2.49	−2.88	−3.75	−2.54	−1.74
8	rapta-B-H_2_O	−4.04	−3.18	−2.16	−3.41	−3.10	−2.40	−3.52	−5.68		−4.10
9	rapta-B-H	−4.01	−2.63	−2.83	−3.03	−4.06	−2.96	−2.81	−4.67	−3.16	−2.56
11	rapta-C	−3.36	−2.34	−2.62	−2.46	−3.31	−1.88	−3.32	−4.18	−2.49	−3.09
12	rapta-C-H_2_O	−3.56	−2.49	−3.35	−2.99	−2.61	−2.61	−3.64	−3.97	−2.37	−2.82
13	rapta-C-H	−3.68	−3.08	−3.44	−2.79	−4.34	−2.66	−3.82	−5.09	−3.00	−3.06
14	rapta-T	−3.25	−2.65		−2.71	−2.39	−2.01	−2.53	−3.91	−2.56	−2.74
15	rapta-Ta-CH_3_	−3.21	−2.19	−2.44	−2.64	−2.25	−2.34	−3.41	−3.74	−2.84	−3.21
16	rapta-Ta-NH_2_	−3.05	−2.56	−3.06	−2.97	−3.96	−2.68	−4.15	−4.76	−2.73	−3.22
17	rapta-Ta-OH	−3.83	−3.16	−3.19	−3.55	−3.24	−3.35	−4.75	−5.02	−2.87	−4.16
18	rapta-T-CF_3_	−3.81	−2.21	−2.58	−2.33	−2.13	−2.31	−3.99	−4.19	−1.91	−2.73
19	rapta-T-CF_3_(H_2_O)	−3.92	−2.35	−2.43	−2.65	−2.47	−2.08	−4.21	−3.95	−2.56	−3.36
20	rapta-T-H_2_O	−3.98	−2.48	−2.61	−2.87	−3.38	−2.23	−3.75	−4.75	−2.57	−3.76
21	rClCOO-NH_3_	−3.18	−2.20	−1.92	−2.95	−3.79	−3.82	−1.96	−2.76	−3.79	−3.19

The results obtained from the docking of these metal-based complexes with the ten chosen receptors using the Gold package are presented in Table 3. The Gold docking results are reported in terms of the values of fitness which means the higher the fitness the better the docked interaction of the complexes, unlike the other two docking packages (Glide and Autodock), which are reported in terms of the docking energy score which means the lower the score the better the interaction. The most targeted receptors from Gold results are accordingly TopII, CatB and kinase. This is in good agreement with the prediction from the Glide package which equally suggested TopII and CatB as part of the most probable targets for the complexes. Those that are averagely targeted are TS, TrxR, rHA and DNA-gyrase. Those targets that are predicted to be least targeted by the complexes are HP-NCP, RNR (except **1** and **5**) and occasionally HDAC7, especially by **1**, **5**, **11**, **12** and **17**. The prediction of CatB as a better target than TrxR is in good agreement with experimental findings [4]. This is not too far from the Glide prediction as HDAC7 was included among the least targeted and others like rHA, TrxR and DNA-gyrase that are here predicted to be averagely targeted are predicted by Glide as least targeted. The complexes that are predicted to bind more strongly to most of the receptors are **3**, **4**, **11** and **16** which also shows the significant effect of the unusual ligand metal complexes, while **1**, **15** and **17** on the average also bind strongly with most of the receptors, but **6** and **21** are predicted to be bind less favourably to most of the receptors. The most likely targets for **1** are RNR and CatB, respectively. By considering the effect of hydrolysis on the binding of the complexes, the results from Gold show that there is a significant improvement in the interaction of the hydrolysed complexes **8**, **20** and occasionally **12** than the parent compounds **7**, **14** and **11**, respectively, just as was predicted by the Glide package. Also in most cases modifications on **14** such as in **15**, **16**, **17** and **18** resulted in increased activities, except in the interaction with HDAC7 and TrxR, which in many cases results in lower activities for the modified forms of **14**.

**Table 3:** The docking prediction for metal-based complexes using Gold.

		CatB	DNA-Gyrase	HDAC7	HP-NCP	Kinase	rHA	RNR	Top11	TrxR	TS
1	carbo-rapta-C	50.37	40.79	−39.09	21.56	39	36.51	51.46	41.13	34.48	42.45
2	rapta-C-COOH	40.53	47.59	30.82	30.46	35.37	33.26	43.02	45.21	38.67	45.48
3	rapta-C-(OH)_2_	45.53	39.29	37.14	21.63	42.04	39.06	38.54	60.73	41.92	52.63
4	rapta-C-NH_2_(OH)	53.29	46.78	39.97	32.2	39.68	43.75	40.18	64.48	40.31	51.22
5	oxalo-rapta-C	40.94	42.48	18.01	25.28	42.51	46.79	46.62	49.95	40.75	40.28
6	raC-NH_2_	32.39	26.92	30.46	25.81	27.59	29.22	24.97	30.63	35.56	30.02
7	rapta-B	45.14	31.96	32.91	22.01	34.92	29.49	28.93	41.53	37.1	34.2
8	rapta-B-H_2_O	45.94	32.76	32.4	26.36	40.14	31.73	29.32	42.14	32.71	31.72
9	rapta-B-H	45.08	32.04	38.21		40.34	33.89		41.4		40.08
10	rapta-B-NH_2_	47.66	37.76	40	28.61	42.27	42.57	36.13	47.9	48.6	38.03
11	rapta-C	44.84	41.05	1.63	24.02	46.02	35.18	27.07	53.56	33.47	38.49
12	rapta-C-H_2_O	40.34	39.56	10.84	26.19	42.19	36.96	36.94	52.18	37.26	37.39
13	rapta-C-H	47.22	39.11	32.49	15.25	43.03	39.26	24.33	51.92	32.93	39.36
14	rapta-T	41.09	34.83	34.43	22.33	38.57	31.1	28.8	46.48	40.48	34.66
15	rapta-Ta-CH_3_	50.09	38.46	38.59	25.11	39.04	35.87	29.64	48.55	30.9	38.84
16	rapta-Ta-NH_2_	50.46	44.05	34.02	30.56	46.61	39.58	41.03	53.8	35.93	38.7
17	rapta-Ta-OH	49.51	43.87	−4.38	28.01	38.15	34.39	42	49.75	36.91	40.75
18	rapta-T-CF_3_	45.33	32.37	33.2	22.83	39.65	30.95	31.99	43.82	37.93	35.02
19	rapta-T-CF_3_(H_2_O)	46.03	27.52	29.51	26.64	43.79	29.8	34.39	45.61	30.34	31.96
20	rapta-T-H_2_O	45.13	33.69	26.55	26.86	44.12	29.71	37.95	46.1	28.8	34.72
21	rClCOO-NH_3_	35.59	38.42	38.51	28.92	32.83	32.54	33.89	40.86	39.64	48.42

The receptors that are recorded as the most targets by many of the metal-based complexes from Autodock prediction (Table 4) are CatB followed by HDAC7, DNA-gyrase, HP-NCP and kinase, except **6** and **7** that bind poorly with most of the receptors, while TS and rHA are predicted as average targets. The predicted least targeted receptor by the complexes is TopII (with the exception of **5**, **17**), RNR and TrxR (with the exception of **21**, **5**, **20**, **4**, accordingly). In all, Glide, Gold and Autodock commonly predicted CatB as one of the most possible targets of the complexes and TrXR as a far less probable target compared to CatB, which is in good agreement with reported experimental results [4]. Also, Glide/Gold included TopII and Gold/Autodock included kinase as one of the best targets. There is a very close relationship in the predictions through the three packages except for the receptors TopII and RNR, which are predicted as rarely targeted by Autodock, but predicted to be one of the most targeted by Glide/Gold and Glide, respectively. There is further agreement between Autodock and Gold as TS, rHA and DNA-gyrase are either predicted as one of the best or an average target for most of the metal-based complexes. The complexes that are predicted to have the best activities against most of the chosen targets are accordingly **1**, **5**, **3**, **2**, **4**, **17**, **8**, **12**, **20**, **10** and **14**. Those metal-based complexes that are predicted to have poor activity against most of the receptors are **6**, **7**, **18** and **21**. Just like the activities of the hydrated complexes are predicted to be highly enhanced using Glide and Gold, so also are **8**, **12**, **20** and **19** predicted to be more active than the respective parent complexes. More specifically, **1** preferentially targets CatB, DNA-gyrase, HDAC7 and TS, respectively, according to the Autodock prediction. The interesting feature of the complexes’ interaction with HP-NCP further supports the experimental finding which suggests HP-NCP as a possible target of rapta complexes [10]. Complexes **3**, **5** and the hydrated complexes **12**, **8**, and **20** bind well with HP-NCP. 

**Table 4:** The docking prediction for metal-based complexes using Autodock.

		CatB	DNA gyrase	HDAC7	HP-NCP	Kinase	rHA	RNR	Top11	TrxR	TS
1	carbo-rapta-C	−9.29	−8.92	−8.33	−3.93	−6.57	−6.49	−5.49	−4.94	−3.04	−8.05
2	rapta-C-COOH	−8.33	−6.63	−7.68	−3.06	−5.51	−5.02	−4.12	−2.63	−5.32	−6.66
3	rapta-C-(OH)_2_	−9.96	−7.9	−8.1	−3.46	−6.65	−5.58	−4	−3.82	−6.43	−6.27
4	rapta-C-NH_2_(OH)	−9.42	−6.79	−7.37	−2.95	−6.16	−5.25	−3.73	−3.1	−6.22	−5.79
5	oxalo-rapta-C	−9.11	−8.35	−7.93	−3.66	−6.44	−5.52	−5.16	−3.8	−7.26	−7.15
6	raC-NH_2_	−3.22	−2.72	−3.69	−2.11	−3.41	−2.96	−2.94	−1.84	−2.78	−2.73
7	rapta-B	−5.01	−3.95	−4.09	−2.61	−3.82	−4.11	−2.65	−2.38	−2.84	−3.89
8	rapta-B-H_2_O	−7.16	−6.21	−6.95	−2.98	−6.3	−4.72	−3.79	−3.22	−5.69	−5.46
9	rapta-B-H	−4.83	−4.77	−5.07	−2.71	−4.81	−4.4	−2.83	−2.42	−3.03	−4.01
10	rapta-B-NH_2_	−7.09	−5.65	−6.76	−2.76	−5.76	−4.23	−3.31	−2.54	−4.24	−4.68
11	rapta-C	−5.73	−5.36	−5.2	−3.08	−4.71	−4.34	−3.46	−3.18	−2.18	−4.65
12	rapta-C-H_2_O	−8.15	−6.58	−6.63	−3.61	−6.21	−5.11	−3.89	−2.78	−5.87	−5.17
13	rapta-C-H	−5.44	−5.14	−5.1	−2.83	−4.69	−4.24	−2.99	−2.65	−3.46	−4.32
14	rapta-T	−7.1	−6.15	−6.23	−2.79	−5.32	−4.62	−3.74	−3.23	−4.67	−5.31
15	rapta-Ta-CH_3_	−5.42	−4.94	−4.49	−3.04	−4.18	−4.48	−3.68	−2.86	−2.64	−4.13
16	rapta-Ta-NH_2_	−5.02	−4.76	−4.37	−2.96	−4	−4.21	−3.11	−2.7	−2.5	−3.77
17	rapta-Ta-OH	−7.15	−7.03	−7.51	−5.14	−6.48	−5.83	−5.32	−3.98	−4.58	−5.99
18	rapta-T-CF_3_	−5.02	−4.59	−4.98	−1.86	−4.33	−3.89	−2.8	−2.04	−1.77	−3.91
19	rapta-T-CF_3_(H_2_O)	−6.57	−5.86	−5.91	−2.31	−5.87	−4.34	−3.07	−2.53	−4.41	−4.76
20	rapta-T-H_2_O	−7.27	−6.26	−6.61	−2.9	−6.32	−5.04	−3.62	−3.23	−4.89	−5.52
21	rClCOO-NH_3_	−5.74	−4.88	−5.57	−2.78	−5.32	−4.81	−3.37	−3.09	−4.25	−3.85

A critical look at the general behaviour of the complexes towards the targets according to the Glide prediction shows that **6** is predicted as the best inhibitor of kinase than any other complexes considered, while **8**, **13**, **17** and **20** are respectively predicted as the best inhibitors of TopII than any other complex. The feature of the Gold prediction shows that **4**, **3**, **16**, **11** and **12** are respectively predicted to have the best activities toward the TopII. Also, **7** and **8** are predicted to best inhibit CatB, while **1** is predicted as the best inhibitor of RNR. The overview of behaviour of the complexes towards the targets from the Autodock prediction shows that **3**, **4**, **5**, **12**, **14**, **20** are the best inhibitors of CatB, while **17** is predicted as the best inhibitor of HDAC7. Therefore, the best inhibitors of TopII from the combined Glide and Gold predictions are **8**, **13**, **17**, **20**, **4**, **3**, **16**, **11** and **12**. The best for CatB from Gold and Autodock combined are **1**, **7**, **8**, **3**, **4**, **5**, **12**, **14** and **20**. This shows that some complexes like **8**, **3**, **4**, **12** and **20** can act as both inhibitors of TopII and CatB together. 

There is a very good agreement between our docked results and the reported experimental behaviour of some of the rapta species as inhibitors of CatB and TrxR [4]. Selecting the rapta complexes that are common with our models, the reported experimental results shows that the inhibitory strength against TrxR starting from the greatest to lowest are in this order: **1**, **5**, **11** and **14**, respectively, with little or no effect of **7** on TrxR. While those of CatB are in this order: **14**, **11** and **1**, respectively, with little or no effect of **5** and **7** on CatB [4]. Also, the inhibition of CatB is predicted to be higher with rapta complexes than TrxR, which is in agreement with the results from all the three packages used. According to the Autodock prediction the inhibition of CatB follows this order: **3**, **4**, **1**, **5**, **12**, **20** and **8**. Also **14** and **11** recorded significant effects, far better than **7**, which is in line with the experimental findings. Compounds **5**, **20**, **17**, **4**, **1**, **8** and **12** are predicted as TrxR inhibitors in that order. The Gold prediction is **1**, **4**, **15**, **16**, **7** and **10** for CatB, while the general behaviours of the inhibitors toward TrxR are very low compared to CatB, just as it was in the reported experimental result. From Glide, the inhibition of CatB by the inhibitors is considered low compared to some other receptors like TopII. However, **20**, **19**, **18**, **8**, **7** are predicted to lead accordingly, while TrxR inhibition is reported to be very poor by most of the complexes, except by **21**. 

In summary, combining the three methods together, just as in the experimental result, **1**, **5**, **12** and **20** are included as part of the best TrxR inhibitor and specifically **1** is rated among the best inhibitors of TrxR by the three docking methods, which is in good agreement with the experimental report. Also, almost all the experimentally found inhibitors of CatB are also included such as **1**, **12** and **20**, except for **5** which are predicted as one of the better inhibitors contrary to the experimental finding. Beyond the agreement of the results for similar complexes with the experimental data, some of our new models of rapta complexes, especially those with unusual ligands, are occasionally predicted to inhibit better than some of the reported common ones.

**Figure 1:**
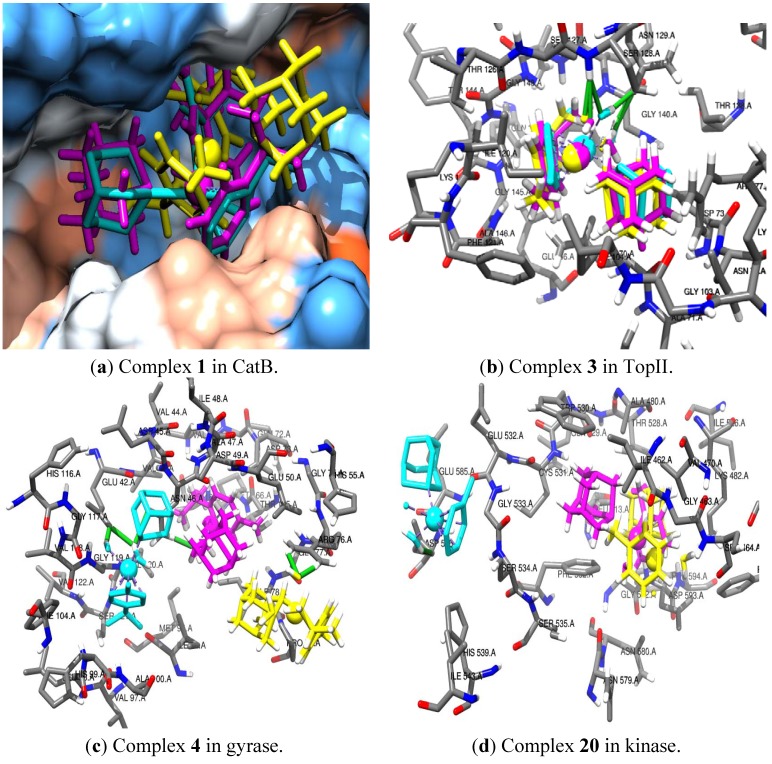
The binding site interaction of complexes **1**, **3**, **4** and **20** with CatB, TopII, gyrase and kinase, respectively, using Autodock (cyan), Gold (magenta) and Glide (yellow) docking predictions. The colouring of the CatB surface as Figure 1a is from the hydrophilic (red) to hydrophobic (blue) and the HB is represented with green cylinders in Figure 1b–d.

The further analysis of the interactions of the Ru(II)-based complexes with the receptors is presented in the Table 5, where the binding site interactions of the first two ranked best complexes for each receptor are shown in terms of the noticeable hydrogen bond (HB) and metal-receptor residue (MR) interactions. Two different binding sites was predicted for kinase, with the first and the second ranked complexes **3** and **1** respectively binding to different sites as in Table 5. Also, the first two ranked complexes **1** and **17** for TopII, respectively, are predicted to bind to two different sites though the second interaction can only be defined by other interactive forces like van der Waals, electrostatic, steric and others since we did not observe any HB and MR. The two best ranked complexes **5** and **12** respectively for TrxR are almost superimposed and the site of their binding is buried inside the receptor. The stronger interaction of **1** with TS (without any noticeable HB and MR) than the complex **5** that is ranked second which has two HB suggests that the metal may not necessarily have a direct interaction with the receptor residues, but just act as a holder of ligands for better and stronger ligand-receptor interaction which may include van der Waals, electrostatic, steric and others. A critical look at the interaction from the Gold prediction as shown in Table 5, further gives insight into the mode of the complex-receptor interaction and the reason while some are ranked as better inhibitors than the others. The binding sites located by the three docking suites are the same for many of the receptors, with that of Gold and Autodock docking specifically in CatB almost being superposed with **1** (Figure 1). The structures with unusual NH_2_ ligands like **10** and **4** which are ranked either first or second as inhibitors of rHA, TrxR and HDAC7, respectively, show that there are very strong metal to receptor residue interactions as the NH_2_ group was seriously pushed off for better direct interaction of the Ru atom with the CH_3_ part of the S(CH_3_) group of MET 87A, arene part of the phenol group of TYR 200 and the arene of PHE 679A, respectively, but in the case where there is an existence of HB between the NH_2_ and receptor as in TopII, where one of the H atoms of NH_2_ and the O atom of the CO group of ASN 129 are interacting through hydrogen bonding, the NH_2_ is well fixed in a correct position with the metal. This suggests that NH_2_ can be a good leaving group where it does not make any HB contribution to the binding of the metal complexes. Another interesting feature which indicates the significant effects of other forms of interactions other than HB and MR that are analyzed in Table 5 is in the case of RNR where **1** that was ranked first has no HB while the second ranked complex **5** as five noticeable HB interactions. In Glide, the first two complexes ranked as inhibitors of rHA, namely **21** and **17**, locate two different binding sites. The presence of more than one binding site observed in our docking studies is not far from the experimental report of multiple binding sites for Ru-(II)-based complexes with HP-NCP [10] and kinase [24].

**Table 5:** The interaction of the binding site residues with first two rank best inhibitors complexes from the three docking methods defining the Complex-receptor existing Hydrogen Bond (HB) and Metal-Receptor (MR) possible interactions with residues within the range of 4.5Å.

No	Method	Receptor Interactions
1	Autodock	CatB^b^{[HB: 1.92 Å (O@COO)-(H@imHIS 111E)], MR:4.07Å (arTRP 221E)};
		Gyrase^a^{[HB:1.74Å (O@COO)-(H@NHVAL 120A)]; [MR:4.35Å (NH_2_ASN 46A)};
		HDAC7^a^{[MR:3.52Å (COOH ASP 626A)]};
		Kinase^b^{[HB:1.36 (O@COO)- (H@imHIS 584A)]};
		rHA^a^{[MR:4.05Å (NH2 ASN 109A)], [MR:4.21Å (COOH GLU 425A)]};
		RNR^a^ {[HB:1.99Å (O@COO)- (H@NHTHR 209A)], [HB:2.55Å (N@PTA)- (H@COOHGLU 441A],}
		TS^a^ {[no HB and MR]}
	Gold	CatB^b^{[MR: 4.32Å (arTRP 221E)]}; RNR^a^ {[MR:3.83Å (HOTHR 209A)]}
3	Autodock	CatB^a^{[HB:1.59Å (OH^1^)-(O@COOGLU 122E)], [HB:1.52Å (OH^2^)-(O@COOH GLU 122E)], [HB:2.52Å (HO^2^)-(H@NH2GLN 23D)], [MR:3.88Å (CH_2_GLY 121E)}; HDAC7^b^{[HB:1.69 (OH)-(O@COOASP 626A)],[MR:4.37Å (arPHE 738A)], [MR:4.39Å (COOH ASP 626A)]; HP-NCP^a^{[HB:2.22 (OH)-(O@COOGLU 64G)],[MR:2.83Å (COOGLU 64G)], [MR:4.19Å (COOGLU 61G)]} Kinase^a^{[HB:1.96Å (H@OH^1^)-(O@COOH ASP 586A)], [HB:2.54Å (H@HO^2^)-(O@COOH ASP 586A)], [HB:3.02Å (O@HO^2^)-(H@COOH ASP 586A)],}
	Gold	Gyrase^a^{[MR:4.48Å (COO GLU 50A)], }
		Top11^b ^{ [HB:2.11Å (O@OH)- (H@NH SER 128A)], [MR:3.97Å (CHSER 127A)], [MR:4.23Å (CO ASN 71A)]}
		TrxR^b^{ [HB:1.73Å (O@OH)-(H@NH2 ARG 166A)], [MR:3.67Å (NH_2_ARG 166A)]}
		TS^a^ {[HB:1.11Å (O@OH^1^)-(H@NH_2_ARG 218A], [HB:2.30Å (O@OH^1^)-(H@OH SER 219A], [HB:2.52Å (O@OH^2^)-(H@NH_2_ARG 23A)][MR:3.47Å (CH_2_ARG 23A)], [MR:3.20Å (NH_2_ARG 218A)]}
4	Autodock	CatB^a^{[HB: 1.44 Å (NH_2_)-(O@COOH GLU 122E)], MR:3.23Å (CH_2_ GLY 29D)};
		Gyrase^b^{HB: 1.87 Å (OH)-(H@NH_2_ ASN 46A)], [MR:2.07Å (CH_2_ASN 46A)], [MR:4.17Å (CH_3_ILE 78A)]}
		HDAC7^a^{[MR:1.87Å (arPHE 679A)], [MR:3.73Å (im HIS 709A) ] }
		TS^b ^{[HB:2.57Å (N@PTA)-(H@OH SER 219A], [MR:2.90Å (SH CYS 198A)], [MR:3.48Å (CH_3_LEU 195A)]}
5	Autodock	HP-NCP^a^{[IT:2.90 (N@PTA)-(O@COOGLU 64G)],[MR:3.73Å (COOGLU 61G)]}
		Gyrase^b^{[HB:1.78Å (O@COO)-(H@NHVAL 120A)], [MR:4.41Å (COGLY 117A)], [MR:4.48Å (CH2GLY 119A)]};
		Top11^a^{[HB:2.08Å (O@COO)-(H@OHSER 128A)], [MR:3.18Å (COOGLU 134A)]}
		TrxR^a^{[HB:1.83Å (O@COO)-(H@NHSER 386A)], [MR:3.96Å (COGLY 38A)]}
		TS^b ^{[HB:1.86Å (O@COO)-(H@SHCYS 198A], [HB:2.22Å (CO)-(H@NH ASP 221A], [MR:4.14Å (CH_2_GLY 225A)]}
	Gold	rHA^a^ {[HB:1.68Å (O@COO)-(H@NH_2_ASN 111A], [HB:2.08Å (O@COO)-(H@NH ASN 111A], [MR:3.20Å (CH2 GLN 33A)]}
		RNR^b ^{[HB:1.86Å (O@COO^1^)- (H@NH GLU 623A)], [HB:1.98Å (O@COO^1^)- (H@NH THR 624A)], HB:2.56Å (O@COO^1^)- (H@OH THR 624A)], HB:1.98Å (O@COO^2^)- (H@OH SER 625A)], [IT:2.81Å (N@PTA)- (O@COPRO 621A], [MR:3.40Å (HOTHR 209A)]}
6	Glide	HP-NCP^a^{[MR:2.42Å (NH3 LYS 113H)]}
		Kinase^a^{[MR:3.01Å (NH ASP 593A) ]}
8	Glide	CatB^a^{[HB:1.96Å (H@H_2_O)-(O@COOGLU 122E)]}
		Gyrase^a^{[HB:1.67Å (H@H_2_O)-(O@COO ASP 49A)]}
		Top11^a^{[HB:1.83Å (H@H_2_O)- (O@OHSER 128A)], [HB:2.04Å (H@H_2_O)- (O@CO ASN 129A)], [IT:2.97Å (N@PTA)- (O@CO ASN 70A)], [MR:3.66Å (NH SER 128A)]} TS^b^{[HB:1.69Å (H@H_2_O)- (H@OHASP 257A)], [MR:3.73Å (OH SER 219A)]}
9	Glide	CatB^b^{[MR:3.09Å (CH_2_ GLY121E)], [MR:4.29Å (COOH GLY122E)]}
		TrxR^b^{[MR:3.42Å OH SER 199A]}
10	Gold	rHA^b ^{ [MR:1.55Å (CH_3_@S(CH_3_) MET 87A)]}
		TrxR^a^{ [MR:4.18Å (ar@ph TYR 200A)]}
12	Autodock	
	Glide	HDAC7^b^{[HB:1.64Å (H@H_2_O)-(O@COOASP 626A)]}
13	Glide	HDAC7^a^{[MR:3.21Å (ar PHE 679A)], [MR:4.42Å (CH_3_ LEV 810A)]}
		Kinase^a^{[MR:3.33Å (ar PHE 582A) ], [MR:3.30Å (CH_3_ILE 462A)]}
		Top11^b^{[MR:4.10Å (CH_2_ASN 70A)], [MR:3.78Å (CH_3_ ILE 104A)]}
15	Gold	HDAC7^b^{[MR:2.80Å (arPHE 679A)], [MR:3.52Å (im HIS 709A) ]
16	Gold	HP-NCP^b^{[HB: 1.69Å(NH2@ar)-(O@COOH GLU 61G)]}
		Kinase^a^{[MR:4.24Å (ar TRP 530A) ], [MR:4.34Å (ar PHE 582A) ] }
17	Autodock	HP-NCP^a^{[HB:2.044Å(OH@ar)-(O@COTHR 101G)]};
		rHA^b ^{[HB:1.88Å (OH@ar)-(O@COPRO 113A], [HB:2.02Å (HO@ar)-(H@NH ARG 145A], [HB:2.11Å (HO@ar)-(H@NH LEU 115A], [HB:2.44Å (N@PTA)-(H@COOH GLU 425A]};
		RNR^b ^{[HB:1.89Å (HO@ar)-(H@NH SER 625A]}
		Top11^b ^{[no HB and MR]}
	Glide	Gyrase^b^{[MR:3.26Å (CH_2_ ILE 78A)], [MR:3.99Å (CH_2_ ASN 46A)]}
		Top11^b^{[HB:1.69Å (OH@ar)- (O@COASP 73A)], [MR:4.33Å (COO GLU 134A)]}
		rHA^b ^{[HB:1.77Å (OH@ar)-(O@CO PRO 110A]}
		RNR^a^{[HB:1.80Å (OH@ar)- (O@OHSER 625A)], [HB:1.86Å (OH@ar)- (H@NHSER 625A)], [MR:4.08Å (H@OH THR 209A)]}
		TS^a^{[HB:2.63Å (OH@ar)- (H@NH_3_ARG 23A)], [MR:4.39Å (NH_2_ ASP 221A)], [MR:3.43Å (SH CYS 198A)]}
19	Glide	RNR^b^{[HB andMR]}
20	Gold	Kinase^a^{[MR:2.83Å (ar PHE 582A) ], [MR:3.46Å (CH_3_VAL 470A)] }
21	Autodock	TrxR^b ^{[HB:1.70Å (H@NH_3_)-(O^1^@COOGLU 341A], [HB:2.32Å (H@NH_3_)-(O^2^@COOGLU 341A)], [HB:1.70Å (H@NH_3_)-(O@COARG 293A], [MR: 3.26Å (COOGLU 341A)], [MR: 3.88Å (COOARG 166A)], [MR: 4.09Å (NH_3_LYS 315A)]}
	Glide	rHA^a^ {[HB:1.74Å (O@COO^1^)-(H@NH_2_ARG 144A], [HB:2.03Å (O@COO^2^)-(H@NH_2_ARG 145A], [HB:1.95Å (O@COO)-(H@OH GLU 141A]}
		TrxR^a^ {[HB:2.62Å (H@NH_3_)-(O@CO VAL 291A], [HB:1.85Å (O@COO)-(H@NH ALA 198A], [MR:3.27Å NH_2_ARG 221A], [MR:3.81Å CH_2_ARG 226A]}

The type of the interaction: MR(Metal-Receptor define for any receptor residue within the range of 4.50Å), HB(Hydrogen Bond Interaction) and IT (interaction predicted to be also HB). The signs im (imidazole group), ar (arene group which in some residues like TRP is part of benzopyrole), @ (part of). The superscript “a” and “b” on each receptors indicate the ranking of the ligand as first and second respectively, while superscript “1” and “2” indicates first and second respectively of the same functional group that exist on a residue, {} separate different receptor, [] separate different interaction in the same receptor while () define the atom with its residue that is involved in the interaction.

The similarity in the three methods of predicting the interaction is also demonstrated by the binding site interaction of complexes **1**, **3**, **4**, **20** with CatB, TopII, gyrase and kinase, respectively, as shown in Figure 1a–d. Taking a critical look at complex **1** in Figure 1a, the cyclobutyl dicarboxylate of the three predictions are towards the boundary of the hydrophilic and hydrophobic part of the CatB, but the arene and the PTA direction of the Glide is different from that of Autodock and Gold. Further agreement is seen in the prediction of Autodock (cyan) and Gold (magenta) as the PTA groups are directed toward an inner hydrophilic large pocket of CatB and the arene groups of the complex are toward the hydrophilic end. The three packages predicted almost the same binding site interaction of complex 3 with TopII residues, as shown in Figure 1b, while there is a difference in the prediction of the interaction of complexes **4** and **20** with gyrase and kinase, respectively. 

Very good agreement has been observed in the structural interaction of the complexes with the receptors using the three methods, but the rankings of the three methods are not the same. On average, there is a better agreement between the ranking of the docked metal-based complexes of the Autodock and Gold (Table 6) with correlation of one of the receptors (*i.e.*, RNR) up to 0.76. The correlation of the Glide with both Autodock and Gold is poor, mainly because Glide did not recognize the Ru atom properly and most of the complexes geometrical orientation on the binding sites of the receptors varies significantly from that of Autodock and Gold, as in Figure 1a. 

**Table 6:** The correlation of the docking results using the Autodock, Gold and Glide packages.

	Autodock *vs*. Gold	Autodock *vs.* Glide	Gold *vs*. Glide
CatB	0.27	−0.50	0.10
DNA-Gyrase	0.54	−0.32	−0.33
HDAC7	−0.13	−0.33	−0.38
HP-NCP	0.24	−0.17	−0.18
KINASE	0.32	−0.17	−0.26
rHA	0.45	−0.37	−0.20
RNR	0.76	−0.32	−0.36
topoII	0.41	−0.06	−0.11
TrxR	0.32	0.13	0.07
TS	0.42	−0.28	−0.46

In order to further understand the properties that are prevalent in determination of the docking score of Glide and the possible causes for the wide difference in ranking from the other two methods, the COMSIA properties was also studied out using the field-based QSAR in Maestro, as shown in Table 7. The major defining factor of the docking score of Glide is predicted to be Gaussian steric property which defines the steric hindrance of the molecule. Another property that defines the docking score of the Glide for the metal-based complexes is the hydrogen bond followed by electrostatic property. The predicted activities agree well with the docked activities based on the statistical properties of significantly high value of R^2^-value ranges from 0.49 to 0.72, high stability ranges from 0.31 to 0.94 and very low P-value ranges from 1.83E−011 to 1.07E−004 (Table 7).

**Table 7:** The statistical results of the QSAR analysis called COMSIA of the Glide docked result.

	Factors	gauss_s	gauss_e	gauss_h	gauss_a	gauss_d	S.D	R^2	R^2-CV	R^2-Scramble	Stability	F	P
CatB	1	0.53	0.15	0.22	0.06	0.05	0.33	0.85	0.41	0.39	0.66	138.8	1.83 E-011
DNA gyrase	1	0.52	0.17	0.19	0.08	0.04	0.53	0.53	0.19	0.39	0.86	27.2	2.41 E-005
HDAC7	1	0.5	0.14	0.19	0.07	0.1	0.42	0.65	0.04	0.45	0.58	41.3	1.82 E-006
HP-NCP	1	0.59	0.14	0.19	0.04	0.03	0.42	0.71	0.35	0.38	0.77	58.5	6.88 E-008
Kinase	1	0.63	0.08	0.16	0.07	0.06	0.68	0.49	0.25	0.35	0.94	21.8	1.07 E-004
rHA	1	0.53	0.16	0.22	0.04	0.05	0.41	0.72	0.02	0.55	0.48	52.9	3.68 E-007
RNR	1	0.63	0.13	0.15	0.05	0.04	1	0.62	0.39	0.45	0.93	38.7	2.00 E-006
TopoII	1	0.43	0.18	0.18	0.07	0.14	0.45	0.65	0.27	0.45	0.72	38.4	3.78 E-006
TrxR	1	0.48	0.15	0.23	0.08	0.06	0.32	0.8	0.03	0.55	0.31	92.6	1.57 E-009
TS	1	0.6	0.16	0.15	0.05	0.03	0.7	0.49	0.27	0.36	0.94	23.4	6.35 E-005

## 3. Experimental

### Computational Methods

The pdb files 1BM0, 2G8D, 4R1R, 3C0Z, 1CSB, 1QZR, 1H6V, 3Q4C, 3MNN and 1AJ6 for the ten receptors: recombinant human albumin (rHA), thymidylate synthase (TS), ribonucleotide reductases (RNR), histone deacetylase (HDAC7), cathepsin B (CatB), topoisomerase II (Top II), thioredoxin reductase (TrxR), BRAF kinase, histone protein in nucleosome core particle (NCP) and DNA gyrase used in this research were obtained from the Protein Data Base (pdb) [37], respectively. The initial preparation of the pdb files to select the needed chains, delete multiple ligands and non-protein parts, molecular graphics and other analyses were performed with the UCSF Chimera package [38]. Three docking suites of programs were used for this study. 

The obtained trial version of Genetic Optimisation for Ligand Docking (GOLD) 5.1 [39] was used for the docking. The Hermes visualiser in the GOLD Suite was used to further prepare the metal complexes and the receptors for docking. The region of interest used for Gold docking was defined as all the protein residues within the 5 Å of the reference ligands that accompanied the downloaded protein complexes except for the protein complex with no accompanied ligand where the binding site was defined from the list of protein residues reported in the literatures to characterise their binding sites. Default values of all other parameters were used and the complexes were submitted to 10 genetic algorithm runs using the GOLDScore fitness function. The second docking suite program used is Autodock 4.2 [40] and its parameter file was modified to incorporate ruthenium metal van der Waals and other needed parameters which were obtained from the Autodock website [41]. The further preparations relevant to Autodock docking were done using the Autodock Tools (ADT) and existing written scripts. The protein and the metal-based structures were prepared by adding Gasteiger charges to each atom of the residues. For the Ru atom charge, we applied the native charge of +2 which is general and can be applied to any complex/receptor interaction study though it may not be as accurate as using an optimized charge which is more specific to the type of the system as it was reported for the docking of the metalloenzymes that contain Zn atom [30]. The three-dimensional affinity and electrostatic grid boxes were generated that cover the entire active site using AutoGrid version 4. The number of grid points in the x, y, z-axes was 60 × 60 × 60 with each point separated by 0.375 Å. Docking calculations were carried with the Lamarkian Genetic Algorithm (LGA). Step sizes of 2Å for translation and 50° for rotation were chosen and the maximum number of energy evaluations was set to 1,750,000. Twenty runs were performed and for each of the 20 independent runs, a maximum number of 27,000 GA operations were generated on a single population of 100 individuals. Default values for the operator weights for crossover (0.80), mutation (0.02) and elitism (1.00) parameters were used. The third suit of program used in this research is the trial version of Glide 5.8 [42] as part of the Maestro 9.3 suite of programs. The Maestro suite was used for all the preparations related to Glide docking. The initial preparation of the metal-based complexes and the receptor were both done using the protein preparation package since the ligand preparation package lacks the necessary force field that could take care of the Ru metal atom. Also, the incomplete receptor residues were corrected using the Prime package [43]. The regions of interest were selected for all the residues around 26 Å of the residue that is selected at the binding sites. The force field used for grid generation is OPLS_2005 and all the default settings for standard precision (SP) were used during the docking. 

Also, further 3D QSAR, specifically COMSIA, was done to know the most significant predictor of the metal-based complexes activities as implemented in the Maestro suite. The correlation table was derived using the statistical tool R [44].

## 4. Conclusions

Through this research, the possible target of some of the metal-based complexes, mostly those of the rapta family have been predicted. It is generally observed that CatB is one of the most possible targets of these complexes as predicted by the three packages. Also, TopII and kinase are predicted by two of the packages as one of the best targets while HP-NCP, HDAC7, DNA gyrase and RNR are suggested by any one of the packages as one of the best targets. Equally, two of the packages predicted RNR as a rare target while TrxR, TS and rHA are either predicted has average or rarely targets of the complexes. This is in good agreement with the reported experimental results on the interaction of rapta complexes with CatB and TrxR, as it was experimentally observed that the inhibition of the TrxR was lower than that of CatB [4]. It is equally interesting to point out the three docking packages clearly show that the activities of the rapta complexes are enhanced when hydrolyzed, which is in good agreement with the experimental reports [3,12,22,36]. Also, the general overview of the selectivity of the complexes shows that the complexes have preferences in the type of the targets they best bind to, which suggests that some of these complexes can possibly be combined together to increase their anticancer activities without any conflict or competition for the same target. Despite all the interesting correlations in the binding modes of the complexes and best possible targets of the complexes, there are manifestations of the deficiencies of the docking packages to predict metal-based complexes compared to their successful application in docking of organic counterpart. Predictions from Autodock and Gold correlate better with experimental data and each other than Glide. The high disparities between Glide and the other docking packages are traced to its bias toward the steric hindrance.
